# Common genetic risk for Parkinson's disease and dysfunction of the endo-lysosomal system

**DOI:** 10.1098/rstb.2022.0517

**Published:** 2024-04-08

**Authors:** Noopur Bhore, Erin C. Bogacki, Benjamin O'Callaghan, Helene Plun-Favreau, Patrick A. Lewis, Susanne Herbst

**Affiliations:** ^1^ Comparative Biomedical Sciences, Royal Veterinary College, University of London, London NW1 0TU, UK; ^2^ Neurodegenerative Diseases, UCL Queen Square Institute of Neurology, University of London, London WC1N 3BG, UK; ^3^ Aligning Science Across Parkinson's (ASAP) Collaborative Research Network, Chevy Chase, MD 20815, USA

**Keywords:** Parkinson's disease, genome-wide association, functional genomics, endo-lysosomal

## Abstract

Parkinson's disease is a progressive neurological disorder, characterized by prominent movement dysfunction. The past two decades have seen a rapid expansion of our understanding of the genetic basis of Parkinson's, initially through the identification of monogenic forms and, more recently, through genome-wide association studies identifying common risk variants. Intriguingly, a number of cellular pathways have emerged from these analysis as playing central roles in the aetiopathogenesis of Parkinson's. In this review, the impact of data deriving from genome-wide analyses for Parkinson's upon our functional understanding of the disease will be examined, with a particular focus on examples of endo-lysosomal and mitochondrial dysfunction. The challenges of moving from a genetic to a functional understanding of common risk variants for Parkinson's will be discussed, with a final consideration of the current state of the genetic architecture of the disorder.

This article is part of a discussion meeting issue ‘Understanding the endo-lysosomal network in neurodegeneration’.

## 1. Parkinson's disease—a complex and heterogeneous disorder

Parkinson's disease (PD) is a progressive and chronic neurodegenerative disorder, first described by James Parkinson in 1817 [1,2]. It is characterized by motor symptoms, such as resting tremor, slowness of movement (bradykinesia), postural instability, gait impairment and limb rigidity [3–5]. There are also substantial non-motor symptoms, including memory and cognitive impairment, apathy, anhedonia, insomnia, fatigue, urogenital issues, dysfunction of the autonomic nervous system and loss of facial expressions. Some of the non-motor symptoms, such as constipation, depression, rapid eye movement sleep behaviour disorder (RBD) and loss of smell (hyposmia), can emerge well before the motor symptoms [6]. The neuropathology of PD is defined by the loss of dopaminergic neurons, predominantly (although not exclusively) within the substantia nigra pars compacta, and by the accumulation of intracellular inclusions called Lewy bodies, primarily made up of an aggregated form of the protein α-synuclein [7,8]. Although PD is characterized by these pathological hallmarks, there is a remarkable heterogeneity in the aetiology and pathogenesis of the disorder [5,9]. The hetereogeneity can manifest as variation in age of onset, disease progression, clinical phenotypes, cellular pathways, neurotransmitter systems, epigenetics and underlying genetic risks [10,11].

It is estimated that over 10 million people are living with PD across the world [12], with males displaying 1.5 times higher risk than females of developing the disease [12]. There are several methodologies to subtype PD depending on genetic, phenotypic or clinical features [13,14]. For example, ageing is a significant factor contributing to the risk of PD and the age of onset itself varies owing to the underlying genetic, environmental and pathophysiological factors. PD can be categorized according to the age of onset as: juvenile- (less than 20 years), young- (21–49 years), middle- (50–69 years) or late-onset PD (greater than 70 years) [13]. Together, the juvenile- and young-onset PD form the early-onset category of PD. The broader spectrum of PD can also be sub-classified as: PD (where movement dysfunction predominates), Parkinson's disease with dementia (PDD), or dementia with Lewy bodies (DLB), according to neurological diagnoses but differing in their secondary symptoms [3]. At present, there are no disease-modifying treatments available, with therapies limited to symptomatic interventions such as levodopa and deep brain stimulation [9].

It is evident that PD is a complex disorder, and the increasing number of people developing the disorder demands a greater understanding of the mechanisms that underlie the aetiology and pathogenesis to provide the foundations for novel avenues of drug discovery.

## 2. Mendelian and monogenic forms of Parkinson's disease

The identification and characterization of genetic variants that cause or predispose individuals to PD has provided important insights into the cellular pathways that result in neuronal death. A pivotal moment was the identification of a mutation (an alanine to threonine coding change at codon 53, A53T) in the *SNCA* gene causative for an autosomal dominant form of PD in 1997, which ushered in a new era of human molecular genetics for PD. *SNCA* encodes α-synuclein, the major constituent of Lewy bodies, and a central player in the aetiology of PD (box 1) [15–20].

Box 1. **α-Synuclein**

α-Synuclein aggregation is one of the most recognizable pathological features of Parkinson's disease (PD). α-Synuclein in complex with other pathological proteins such as tau or amyloid-β along with lysosomes, lipids and other proteins forms pathological lesions known as Lewy bodies (box figure (*a*)) [21,22]. The presence of Lewy bodies in the brainstem or the limbic region is a hallmark of PD, and the progression of those lesions to the neocortex defines PDD and DLB; however, some heterogeneity exists in disease manifestation owing to additional genetic or neuronal factors [23]. Despite its clear pathophysiological role in PD, the physiological function of α-synuclein is still debated. α-Synuclein can bind lipid membranes and sense their curvature, which has led to the hypothesis that it plays a direct role in synaptic vesicle trafficking and neurotransmitter release [24]. Additionally, α-synuclein has been reported to be involved in transcription and translation [25], and plays a role in the fission and fusion of mitochondria in nigrostriatal dopaminergic neurons [26]. Further, synergistic interactions have been suggested between α-synuclein, dopamine and calcium in nigral and locus coeruleus neurons [27].

At a genetic level, polymorphisms in the *SNCA* promoter are associated with idiopathic PD. Gene dosage also plays an important factor in *SNCA* pathology, with gene duplications and triplications reported in PD and DLB—with the severity of disease displaying a dose dependency [10]. Pathological coding mutations in the *SNCA* gene have been reported in PD, including A30P, G51D, A53T and E46K, with variants increasing the propensity of α-synuclein to aggregate (box figure (*b*)). α-Synuclein abnormalities have been associated with mitochondrial dysfunction such as mitochondrial DNA fragmentation, decreased protein import, decreased ATP production, and increased reactive oxygen species (ROS) production, as well as endo-lysosomal dysfunction. This includes impairment of several steps in the endo-lysosomal machinery, thus impacting the autophagic pathway and lysosomal degradation, and disruption of synaptic vesicle transport. α-Synuclein dysfunction also leads to alteration in the retromer trafficking and toxic aggregates in the form of cytoplasmic inclusions through the endo-lysosomal pathway. One of the most prominent theories of α-synuclein aggregation is its spread from neuron to neuron in a prion-like propagation, supposedly through endosome or through tunnelling nanotube formation [20].

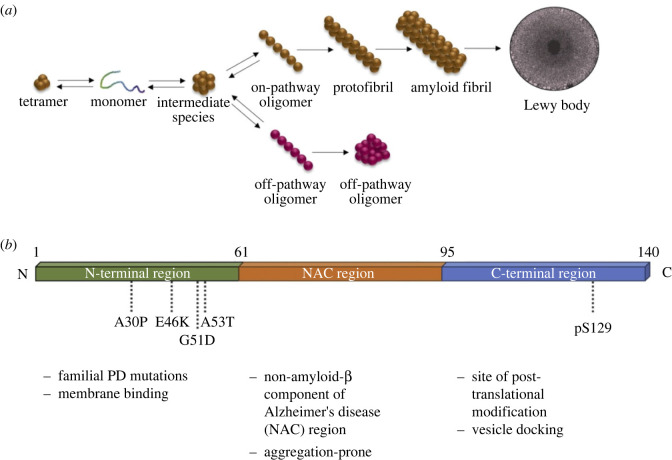

Prior to this, PD was considered a prototypic non-genetic form of neurological disorder—occurring sporadically or spontaneously [28–30]. Since then, substantial advances have been made with regards to the identification of monogenic forms of Parkinson's disease, spanning autosomal dominant, autosomal recessive and X-linked patterns of inheritance [28–30]. The monogenic forms of PD may not always follow classical Mendelian inheritance, and may present with varying ages of disease onset, depending on the gene studied. Monogenic loci can be further subtyped according to the clinical phenotypes such as tremor-dominant, akinetic-rigid, postural instability, gait-difficulties, mixed, or indeterminate subtype, depending on the primary symptomatic predispositions [13,31]. They may also be classified according to the progression of motor and non-motor symptoms, and it is apparent that auxiliary genetic or environmental factors may affect disease causation in monogenic PD. A summary of monogenic variants associated with Parkinson's disease is shown in table 1.

**Table 1.** Monogenic Parkinson's disease genes. GWAS, genome-wide association studies; JOPD, juvenile-onset PD; YOPD, young-onset PD; MOPD, middle-onset PD; LOPD, late-onset PD; AD, autosomal-dominant; AR, autosomal-recessive; GOF, gain-of-function; LOF, loss-of-function; probable, higher likelihood/orthologue evidence; possible, high likelihood/associative evidence; ER, endoplasmic reticulum, EGFR, epidermal growth factor receptor. *Same *PRKN* locus.

no.	gene symbol	gene name	age of onset	inheritance pattern	GWAS hit	confidence as PD gene	mechanism	proposed cellular functions
1	*SNCA*	*synuclein-alpha*	YOPD or MOPD	AD	yes	very high	LOF/GOF	synaptic vesicle trafficking, molecular chaperone activity [32,33]
2	*LRRK2*	*leucine-rich repeat kinase 2*	LOPD	AD	yes	very high	GOF	vesicle trafficking [34]
3	*GBA1*	*glucosylceramidase-beta 1*	YOPD	AR	yes	very high	LOF/GOF?	lysosomal ceramide metabolism [35]
4	*VPS13C*	*vacuolar protein sorting 13 homologue C*	YOPD	AR	yes	very high	LOF	lipid transfer from ER to lysosomes [36]
5	*VPS35*	*VPS35 retromer complex component*	LOPD	AD	no	very high	LOF	endo-lysosomal sorting [37]
6	*ATP13A2*	*ATPase cation transporting 13A2*	YOPD	AR	no	very high	LOF	lysosomal polyamine transporter [38]
7	*PRKN*	*Parkin RBR E3 ubiquitin-protein ligase*	YOPD or JOPD	AR	no	very high	LOF	mitochondrial quality control [39]
8	*PINK1*	*PTEN-induced kinase 1*	YOPD	AR	no	very high	LOF	mitochondrial quality control [39]
9	*DJ-1*	*parkinsonism associated deglycase*	YOPD	AR	no	very high	LOF	involved in oxidative stress, mitochondrial quality control [40]
10	*PLA2G6*	*phospholipase A2 group VI*	YOPD	AR	no	very high	LOF	membrane lipid biosynthesis [41,42]
11	*FBXO7*	*F-box protein 7*	YOPD	AR	no	very high	LOF	E3 ubiquitin-protein ligase [43]
12	*CHCHD2*	*coiled-coil–helix–coiled-coil–helix domain-containing 2*	LOPD	AD	no	very high	both	hypoxia-responsive transcription factor; mitochondrial electron transport [44]
13	*GCH1*	*GTP cyclohydrolase 1*	YOPD	AD	yes	very high	LOF	involved in neurotransmitter synthesis, nitric oxide synthesis, GTP binding [45,46]
19	*LRP10*	*LDL receptor-related protein* 10	YOPD or MOPD	AD	no	very high	LOF	lipoprotein uptake and endosomal sorting [47,48]
14	*DNAJC6*	*DnaJ heat shock protein family (Hsp40) member C6*	YOPD	AR	no	high	LOF	uncoating of clathrin-coated vesicles [49]
15	*SYNJ1*	*synaptojanin 1*	YOPD	AR	no	high	LOF	synaptic vesicle phosphoinositide metabolism [50]
16	*ATP10B*	*ATPase phospholipid-transporting 10B*	YOPD	AR	no	high	LOF	lipid transfer from ER to lysosomes [51]
17	*DCTN1*	*dynactin subunit 1*	YOPD or MOPD	AD	no	high	LOF	transport along microtubules [52]
18	*POLG*	*DNA polymerase-gamma, catalytic subunit*	YOPD	AR and AR	no	high	LOF?	replication of mitochondrial DNA [53]
20	TMEM230*	*transmembrane protein 230*	LOPD	AD	no	low	LOF	involved in trafficking and recycling of synaptic vesicles [54]
21	DNAJC13*	*DnaJ heat shock protein family (Hsp40) member C13*	LOPD	AD	no	low	GOF	endosomal membrane tubulation and trafficking [55]
22	*PSAP*	*prosaposin*	MOPD or LOPD	AD	no	low	LOF?	lysosomal sphingolipid metabolism [56]
23	*HTRA2*	*HtrA serine peptidase 2*	YOPD or LOPD	AD	no	low	LOF	serine protease activity, response to oxidative stress, apoptosis [57]
24	*EIF4G1*	*eukaryotic translation initiation factor 4 gamma 1*	LOPD	AD	no	low	LOF	ER stress-dependent translation [58]
25	*GIGYF2*	*GRB10-interacting GYF protein 2*	LOPD	AD	no	low	undetermined	component of translation initiation repressor complex [59]
26	*SCA2*	*ataxin 2*	YOPD or MOPD	AD	no	undetermined	LOF?/GOF?	involved in EGFR trafficking, stress granule formation, regulation of translation [60]

**Figure 1.**
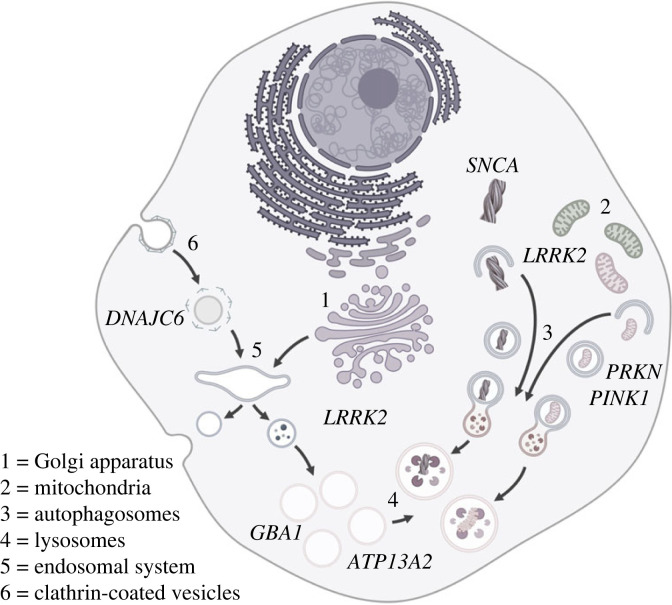
Endo-lysosomal pathways and monogenic Parkinson's disease. Parkin and Pink1 (*PRKN* and *PINK1*) play a central role in mitochondrial quality control. LRRK2 and α-synuclein (*LRRK2* and *SNCA*) have been implicated in the regulation of macroautophagy, with LRRK2 also linked to vesicle trafficking at the trans-Golgi and lysosomal damage response. Glucocerebrosidase and ATP13a2 (*GBA1* and *ATP13a2*) have roles in maintaining lysosomal function. Auxilin (*DNAJC6*) acts to uncoat clathrin-coated vesicles during clathrin-mediated endocytosis.

The identification of monogenic forms of PD has provided the foundations for functional characterization of the biological changes that result in neurodegeneration, studies that have revealed several common pathways. These include, but are not limited to, mitochondrial dysfunction, protein aggregation and disruption of the endo-lysosomal system, portrayed and summarized in figure 1 and table 1, and reviewed in the references cited therein and in [5,61].

## 3. Common genetic risk for idiopathic Parkinson's disease

**Figure 2.**
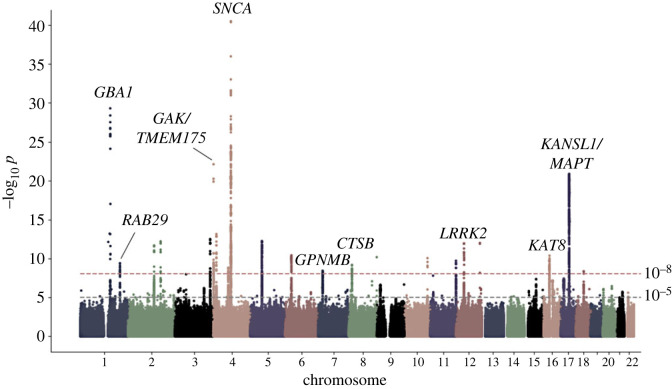
A Manhattan plot for genome-wide associated variants in Parkinson's disease, showing –log_10_*p* for single nucleotide polymorphisms across the autosomes. The horizontal dotted lines indicate the corrected thresholds for achieving genome-wide significance, with example candidate genes for loci discussed in this review indicated [68].

Monogenic PD represents a minority of cases—estimated to be about 1 in 10, with some variation depending on the population studied [62,63]. The overwhelming majority (>90%) of people living with Parkinson's do not have a single genetic cause of their disease, and the overlap between the functional biology of monogenic and idiopathic disease has only recently begun to emerge [12]. This has been driven by advances in genomic technology, reducing the cost of extensive genetic analysis of large numbers of individuals, and facilitating genome-wide association studies (GWAS) for idiopathic PD, in order to identify common genetic risk for the disease [64,65]. These analyses identify allelic variants that modulate the likelihood of a particular phenotype [66]. Several large-scale GWAS have now been carried out for risk of Parkinson's disease, with the most recent, published in 2019, presenting a meta-analysis of over 30 000 cases and 1 000 000 controls [67]. This identified dozens of loci across the human genome significantly associated with the risk of disease (frequently projected as a Manhattan plot, as shown in figure 2). With rapidly increasing numbers of participants, additional studies have been carried out moving beyond absolute risk of disease to assess genomic variation influencing age of onset, progression, motor subtypes, and risk of dementia, as well as providing insights into multi-ethnic heterogeneity in risk [69–73]. Together, these are beginning to map out how an individual's genetic background influences the likelihood of developing PD, and what happens following the onset of symptoms.

## 4. Challenges of understanding genome-wide association at a molecular level

**Figure 3.**
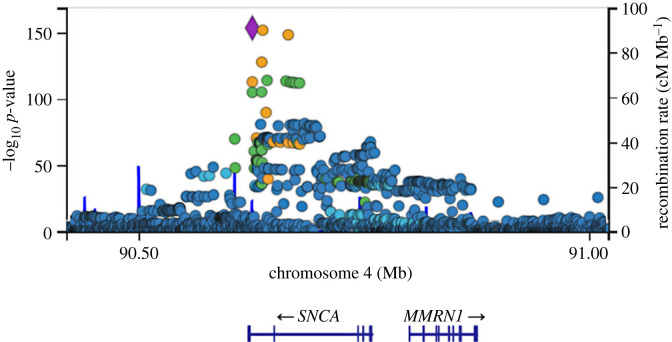
The *SNCA* locus on chromosome 4 and genome-wide associated single nucleotide polymorphisms. Data are derived from the Meta Five genome-wide association analysis, and accessed through the IPDGC genome browser [77].

The data emerging from GWAS are complex and represent a starting point for translating genetic risk for PD into functional insights rather than being a functional end point in and of themselves. First, in the case of PD, the impact of each individual associated locus is relatively modest, with a small increase in overall risk—to the point where taken individually there is negligible predictive power. It is only when polygenic risk is considered, merging multiple common risk factors, that anything approaching clinical relevance can be arrived at [74,75]. Secondly, moving from the identification of associated loci to pinpointing the biological changes driving that association is a significant challenge [76]. The initial issue is the task of nominating a gene or genes as candidates underlying the association. In some cases, this might be relatively straightforward, with only one gene falling under the significantly associated single nucleotide polymorphisms and/or with a clear functionally impactful coding variant within the candidate gene. In many cases, however, there may be multiple genes falling under the association peak at a genome-wide significant locus. Of the loci identified in the most recent meta-analysis for genome-wide association in Parkinson's disease, only a small fraction of genes under the association peaks presented with conspicuous functional links to previously identified Mendelian forms of PD. For one example, in the *SNCA* gene on chromosome 4 (figure 3), the association is driven by non-coding variation regulating expression, distinct from the coding or copy number structural variants observed in the Mendelian form of the disease [78,79]. Notably, the majority of genes in the Nalls *et al*. study [67] were nominated based upon proximity to the sentinel single nucleotide polymorphism (that is, the polymorphism with the most significant association) for the locus. Identifying the gene driving association, and defining the mechanisms underlying association are, therefore, a non-trivial undertaking—and one that will require substantial efforts to complete [80]. An example of how to approach this is provided by Kia and co-workers, who applied analysis of expression, epigenetic modifications, and protein networks to triage and nominate likely candidates emerging from the 2019 GWAS meta-analysis across the genome in PD [81]. There are limitations to such an approach, for example not having accounted for epistasis or pleiotropy of the analysed genes, besides assumptions that the true causal variant is being triaged with certainty [81]. One possible way to overcome this challenge is by using the Mendelian randomization (MR) method, which can draw inferences on the effect of the genetic risk variants, thus possibly providing a better prediction of causation, based on stronger statistical powers [82,83].

Additionally, the majority of current GWAS studies have been based upon data derived from Western cohorts [30]. This issue is being mitigated by more inclusive studies and global initiatives like the Global Parkinson's Genetics Program, focusing on diverse cohorts. The novel hits derived from such studies underscore the heterogeneity of PD in different populations. For example, Rizig and coworkers identified a *GBA1* risk variant in African and African-admixed PD cohorts that is distinct from previously identified *GBA1* risk alleles [84], Foo and coworkers found *SV2C* and *WBSCR17* as novel hits, along with *GBA1*, *TMEM175*, and *LRRK2* etc., in East Asian cohorts [85], an Indian study found hits such as *SNCA*, *TMEM175*, *GBA1*, *PRKN* and *BSN* [86], and a multi-ethnic study including Latin and Latin-admixed populations identified *SNCA*, *STXBP6* and *RPS6KA2* as novel loci in their meta-analysis [87]. Interestingly, the effect of sample size is further demonstrated by Grenn and coworkers, who found *GBA1* as a risk factor in European populations only after using larger sample size obtained from diverse datasets [77].

Furthermore, the prevalence of idiopathic PD has been found to be higher in males than in females, whereas there has been no gender bias observed in monogenic forms of PD, such as in LRRK2 G2019S mutation carriers. Recent GWAS studies have explored the causation behind gender differences in European cohorts. However, they have been unable to find any conclusive argument from the analyses of the autosomal and sex chromosomes [88–90]. A comprehensive meta-analysis of several diverse cohorts also did not offer any leads on gender differences [91]. Similar findings were observed in a recent study, where gender differences in *GBA1* variants could not explain PD risk but predicted a stronger association of males with DLB [92]. In summary, there are several challenges associated with the molecular dissection of Parkinson's disease GWAS, which can only be resolved by larger and inclusive studies, with refined analyses. Until then, caution needs to be exercised before drawing definitive conclusions.

## 5. Pathways to parkinsonism—mitochondrial and endo-lysosomal dysfunction

Intriguingly, even with our incomplete knowledge of the genes underlying genome-wide association, there is evidence of convergence between the monogenic and common risk variants found in PD. There are a number of genes that are found in both aspects of genetic risk for Parkinson's disease—*SNCA*, as outlined above, but also *LRRK2* on chromosome 12 and *GBA1* on chromosome 1 (see table 1) [93,94]. This overlap implies shared biology between monogenic and idiopathic PD, and reinforces a role for specific cellular pathways in the pathogenesis of PD. Genes highlighted by GWAS and underlying monogenetic risk point to the endo-lysosomal system and mitochondrial quality control as two distinct, but overlapping, pathways leading to PD [95,96]. The endo-lysosomal system is formed by dynamic membrane-bound structures that are involved in processes such as endocytosis, phagocytosis and autophagy to carry out complex functions like macromolecule sorting, cellular signalling, proteostasis, organelle homeostasis and membrane organization. The terminal organelle, the lysosome, is a primary catabolic organelle that has the capacity to break down aggregated proteins or even entire organelles, such as mitochondria. It also serves as a signalling hub that communicates information about amino acid availability or proteotoxic stress to the rest of the cell. Mitochondria, in contrast, are the main producers of cellular energy but also ROS. As ROS can be damaging to the cell and mitochondria themselves, a functioning mitochondrial quality control system is vital in maintaining cellular health. The Parkinson's disease-associated cluster of proteins implicated in the endo-lysosomal system includes the lysosomal β-glucosylceramidase (encoded by *GBA1*), the lysosomal polyamine exporter ATP13A2 [97], the endoplasmic reticulum (ER)-to-lysosome lipid transfer protein VPS13C [98,99], the lysosomal proton channel TMEM175 [100] and VPS35, which is part of the retromer complex involved in endo-lysosomal sorting of proteins (table 1 and figure 1). Of note, some of these candidates, e.g. *VPS13C*, have been identified in monogenic forms of PD and via GWAS. Together, these proteins highlight deficits in lysosomal metabolic processes and sorting events along the endo-lysosomal pathway as disease-causing in PD. On the other hand, PINK1 and PRKN are key players in the mitochondrial quality control system by instigating the removal of damaged mitochondria via autophagy. Additionally, CHCHD2 and VPS13C can impact mitochondrial quality control in a PINK1/PRKN-dependent manner [101–103]. Notably, perturbations of genes influencing the endo-lysosomal system may also affect mitochondrial quality control and *vice versa*. Lysosomal dysfunction will impact mitochondrial quality control as fusion with the lysosome is the final degradative step in the autophagic removal of organelles, and organelle cross-talk, e.g. via lysosome–mitochondria contact sites, impacts the function of each organelle [104].

However, the task of defining the candidate genes at loci identified through studies for Parkinson's disease is still ongoing. To illustrate some of the challenges it presents, and how novel associations are expanding a role for the endo-lysosomal system and mitochondrial dysfunction in Parkinson's disease, two case studies are presented here—examining the loci at chromosomes 7p15.3 and 16q11.2/17q21.

## 6. Chromosome 7p15.3, *GPNMB* and lysosomal function

**Figure 4.**
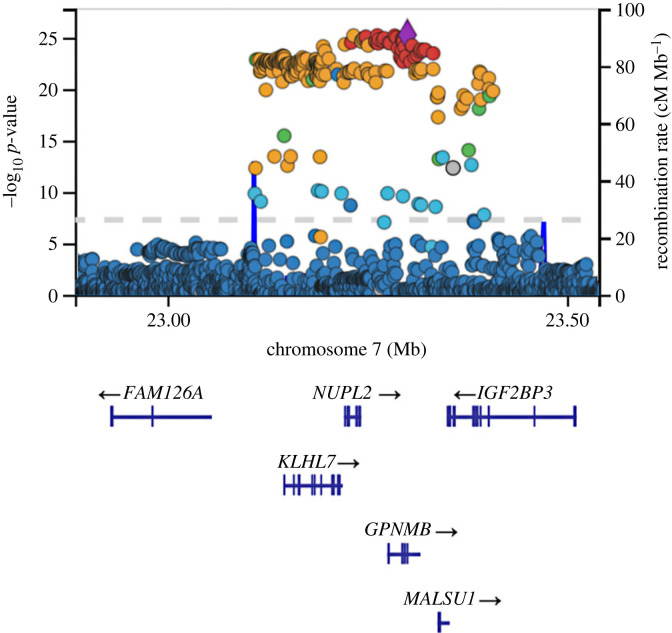
The chromosome 7p15.3 locus and *GPNMB.* Significant single nucleotide polymorphism (SNPs cover a range of genes, including *NUPL2*, *KLHL7* and *GPNMB*. The most significant SNP is located in a *GPNMB* intronic region. Data are derived from the Meta Five genome-wide association analysis, and accessed through the IPDGC genome browser [77].

The association at chromosome 7p15.3 was first identified as a Parkinson's disease risk-associated allele through a two-stage meta-analysis as an extension of a previously conducted GWAS [105]. This association occurs as a non-coding A to G change at rs199347 on chromosome 7 [106], with three candidate genes (*KLHL7, NUPL2* and *GPNMB*) in linkage disequilibrium falling under the association peak (figure 4). To discriminate between these three candidate genes, an expression quantitative trait locus analysis was carried out, indicating the risk allele is associated with increased brain expression of *GPNMB*, but not *KLHL7* or *NUPL2* [107,108]. Coincident with this, the sentinel single nucleotide polymorphism sits within the *GPNMB* locus. These data, therefore, support increased expression of *GPNMB* in brain tissue as being associated with heightened PD risk at this locus. It is worth noting, however, that this does not exclude a role of *KLHL7* or *NUPL2*, as additional transcript-specific expression quantitative trait loci (eQTLs) at this locus are associated with differential expression of both genes in immune tissues [108]. This indicates a potentially complex function of this eQTL, and highlights the need to consider the roles that several differentially expressed genes can play in different cell types and tissues, and at different disease stages.

GPNMB has been implicated in melanosome formation, autophagy, inflammation and various human diseases. Specifically, increased GPNMB expression has been linked to poorer prognosis in breast cancer patients [109], and mutations in the *GPNMB* gene have been named as causal of the skin pigmentation disorder amyloidosis cutis dyschromica (ACD) [110]. Since the discovery of the association between *GPNMB* and Parkinson's disease, several studies have observed GPNMB function in the context of neurodegeneration and models of PD-related pathology. Increased *Gpnmb* transcription has been observed in rodent models of PD [111], while post-mortem brain tissues of PD patients display increased GPNMB protein, specifically within the substantia nigra [112]. Moloney and co-workers observed raised GPNMB levels in the context of both α-synucleinopathy and lipidopathy. While elevated α-synuclein levels showed no effect on *Gpnmb* expression, inhibition of glucocerebrosidase activity (and therefore induction of lipidopathy) resulted in marked increases in GPNMB expression, implying a role of GPNMB in the context of lipid accumulation and lysosomal dysfunction. Additional work, motivated by the linkage of increased GPNMB expression and PD risk, hypothesized a protective effect of *Gpnmb* ablation in various mouse models of neurodegeneration, but found no effect of *Gpnmb* knockout when compared with wild-type mice [113]. By contrast, a recent study functionally implicates GPNMB in the cellular uptake of α-synuclein, giving rise to the possibility that GPNMB aids the spreading of α-synuclein throughout the brain in PD [107]. Intriguingly, there is evidence accruing to support a role for GPNMB in lysosomal integrity—although how these data relate to a putative role for GPNMB in PD is, to date, unclear [114,115]. Taken together, there is an emerging yet still incomplete picture of GPNMB's involvement in PD pathology, especially regarding the endo-lysosomal system. Indeed, most of GPNMB's proposed involvement in lysosomal function has been the result of observations made in lysosomal storage disorders [116–118]. Van der Lienden *et al.* [118] describe GPNMB as an emerging biomarker for lysosomal dysfunction, but its cellular and molecular role in disease mechanisms remains elusive [118]. While strides have been made in our understanding of GPNMB's role in PD, evidence suggests it performs a complex function in PD pathobiology and the endo-lysosomal system, necessitating further investigation.

## 7. Chromosomes 16q11.2/17q21, *KAT8*/*KANSL1* and mitophagy

**Figure 5.**
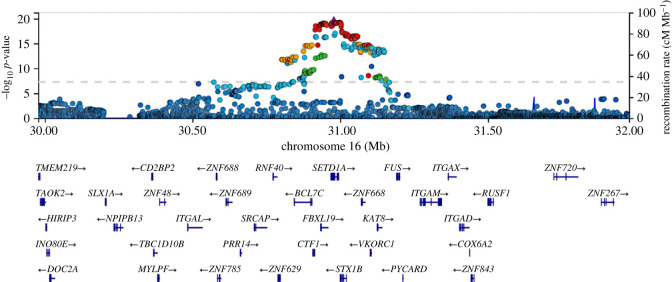
The chromosome 16q11.2 locus and *KAT8*. The signal at 16q11.2 covers multiple genes, including *SETD1A*,** which was originally nominated as the lead single nucleotide polymorphism, and *KAT8*, which has been nominated through functional cell-based screening. Data are derived from the Meta Five genome-wide association analysis, and accessed through the IPDGC genome browser [77].

Mechanistic insight into the association at 16q11.2 emerged from an unbiased functional screening approach. Based on the hypothesis that idiopathic and Mendelian PD share common underlying disease pathomechanisms, Soutar and co-workers sought to investigate whether Parkinson's disease risk GWAS candidates regulate the PINK1/Parkin-dependent mitophagy process [119]. In this study, 36 PD risk GWAS candidate genes from significant risk loci reported in the 2017 PD GWAS were first prioritized through a triage process applying bioinformatic strategies [67,120,121]. Phenotypic screening and a primary validation of prioritized PD GWAS risk genes, revealed *KAT8* to be a novel regulator of the PINK1/Parkin-dependent mitophagy process, with *KAT8* knockdown leading to a reduction in phospho-ubiquitin (Ser65) deposition. The *KAT8* gene is located within the 16q11.2 risk locus (figure 5), although, as shown by the association signal at this locus, there are many potential candidate genes. KAT8 is a lysine acetyltransferase that represents the catalytically active subunit of the non-specific lethal (NSL) epigenetic remodelling complex, which is responsible for the deposition of pro-transcriptional histone H4 acetylation modification [122]. The NSL complex has been shown to be associated with organism development, cellular homeostasis, and mitochondrial DNA transcription [123–125]. Specifically, KAT8 is involved in cerebral and neural stem cell development, and its variants have been linked to intellectual disability, epilepsy and autism [126]. Soutar *et al*. showed that knockdown of several other components of the NSL complex also lead to impairments in Ser65 phospho-ubiquitin deposition, including the NSL complex member *KANSL1*, which is itself another PD GWAS candidate risk gene [119,127]. *KANSL1* is located within the 17q21 PD risk locus, which is in linkage disequilibrium with the commonly occurring *MAPT* H1 haplotype [119,127,128]. Besides this, genetic changes in *KANSL1* play a causative role in Koolen–de Vries syndrome in children, which is characterized by intellectual disability, epilepsy, neuromotor phenotypes and other neurocognitive abnormalities [129]. KANSL1 and KAT8 KD were both shown to reduce the mitochondrial accumulation of PINK1 upon mitochondrial membrane depolarization, and consequently downstream PINK1-dependent steps of the mitophagy cascade. Given the canonical function of the NSL complex as a pro-transcriptional epigenetic remodelling complex, impairments in *PINK1* gene expression represented a strong candidate mechanism accounting for the PINK1-deficits observed. In line with this hypothesis, the authors showed reduced *PINK1* mRNA levels following both *KANSL1* and *KAT8* siRNA knockdown. Of relevance, eQTLs and allele-specific expression (ASE) analysis linked PD risk at these two loci with reduced *KANSL1* and *KAT8* expression [130,131], in line with the phenotypic data highlighting impaired PINK1-dependent mitophagy initiation. In addition to PINK1 mitophagy initiation, KANSL1 and KAT8 have also been shown to play an important role in regulating the expression of autophagy-related genes, autophagy and lysosomal function, further implicating the NSL complex in endo-lysosomal regulation, beyond PINK1 mitophagy alone [123,132,133].

## 8. Constructing a genetic architecture for Parkinson's disease

**Figure 6.**
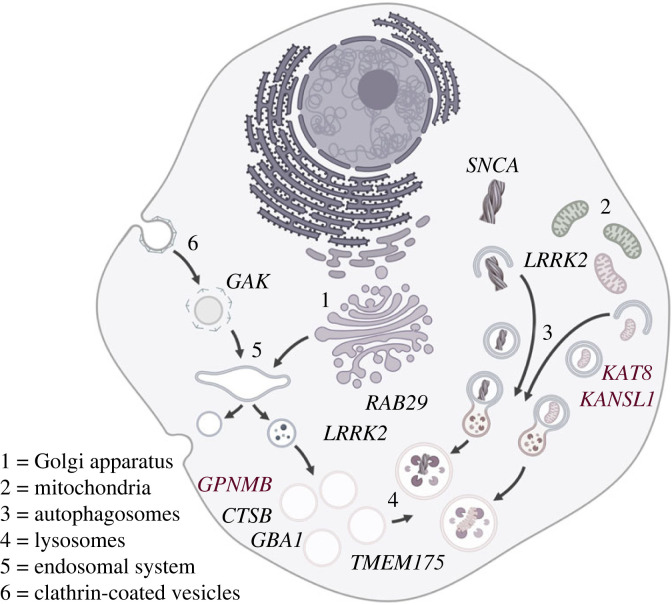
Endo-lysosomal pathways and genes implicated by genome-wide association studies for Parkinson's disease. KAT8 and KANSL1 (*KAT8* and *KANSL1*) have been implicated in the regulation of mitochondrial quality control through mitophagy. As for monogenic associations, LRRK2 and α-synuclein (*LRRK2* and *SNCA*) have been implicated in the regulation of macroautophagy, with LRRK2 and Rab29 (*RAB29*) also linked to vesicle trafficking at the trans-Golgi and lysosomal damage response. Glucocerebrosidase *(GBA1*)*,* cathepsin B (*CTSB*), GPNMB (*GPNMB*) and TMEM 175 (*TMEM175*) are all linked to catabolic lysosomal function. Finally, GAK (*GAK*) plays an analogous role to *DNAJC6* in uncoating clathrin-coated vesicles.

These two examples are functionally congruent with the evidence from a number of other genome-wide associated candidate genes as contributing to dysregulation of endo-lysosomal function in idiopathic PD, mapping onto that observed in monogenic PD (figure 6).

**Figure 7.**
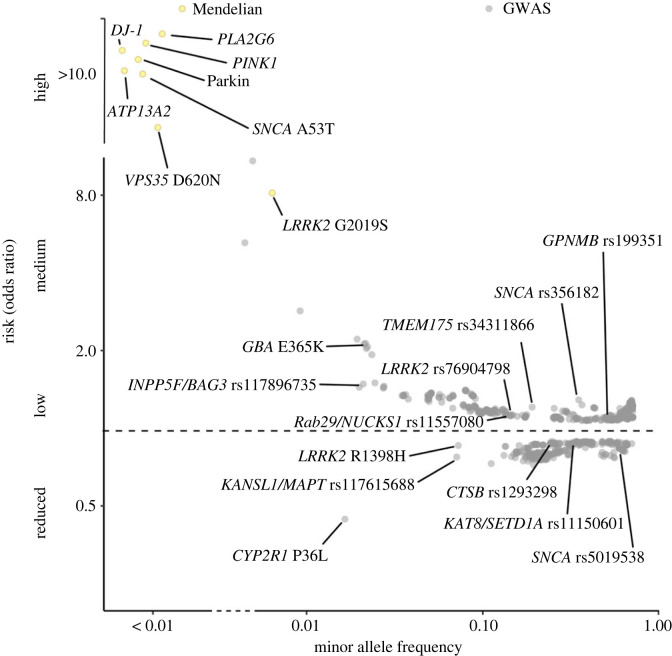
The emergent genetic architecture of Parkinson's disease, showing monogenic and genome-wide associated loci. Odds ratios and population frequencies are derived from the IPDGC genome browser [77].

Building on the insights from GWAS, it is now possible to move beyond the dichotomous divide between monogenic and idiopathic (including polygenic) forms of the disorder and start to construct a comprehensive genetic architecture for PD. There is substantial evidence for a continuum of risk, quantified as altered odds ratio for disease, for several of the genes involved in monogenic Parkinson's disease, such as *SNCA*, *LRRK2* and *GBA1* (all with direct or close links to the endo-lysosomal system). These can be integrated into an increasing number of more common variants in strong candidate genes for increased risk of disease—as well as a smaller number of variants associated with lowered odds ratio and therefore decreased risk of disease (figure 7). This architecture has important implications for our understanding of PD. It provides evidence for shared aetiology between monogenic and idiopathic PD, while simultaneously highlighting the breadth of the disease pathways involved in the disorder. A key consequence of the former is that it increases the likelihood of drugs being developed to target monogenic PD, such as those linked to α-synuclein and LRRK2 forms, having relevance to the wider population of people living with the disease. Several of these, such as the antibody therapies aiming to remove α-synuclein from the brain, or small molecule kinase inhibitors of LRRK2, are currently undergoing clinical trials (NCT04777331, NCT05424276, NCT05670782, NCT03976349, NCT03710707, NCT04056689) [134–136]. Importantly, the identification of pathways linked to disease risk, beyond individual genes and proteins, also opens the door to targeting processes rather than individual genes—expanding the spectrum of drug discovery in Parkinson's.

## 9. Conclusion and future perspectives

Taken together, the past two decades have borne witness to substantial advances in our understanding of the causes of PD, with much of this deriving from enhanced comprehension of the molecular genetics of the disorder. However, much remains to be done. Most obviously, the functional changes underlying the majority of genome-wide associated loci are obscure—information that has the potential to provide even greater insight into the events that precede and drive neurodegeneration in the PD brain. The increasing application of multi-omic analysis, alongside detailed mechanistic characterization, provides hope that many more genome-wide associated loci will be clarified in the coming years. It is also clear that efforts to increase the diversity of genetic analyses of Parkinson's will yield important novel insights into the genetic architecture of the disease. Coupled to more sophisticated stratification of patient populations and moving beyond absolute risk to disease modifiers and progression, there will undoubtedly be more genetic information feeding into functional investigations—including those targeting mitochondrial quality control and the endolysosomal system. Although categorizing risk genes into functional categories can help to focus research efforts, assigning GWAS risk genes requires unbiased approaches and an open mind in order not to overlook novel avenues to understanding and treating PD. Crucially, insights deriving from these studies will aid in defining molecular sub-types of Parkinson's and support the development of novel drug targets for this disorder.

## Data Availability

This article has no additional data.
